# Passive leg raising test versus rapid fluid challenge in critically ill medical patients

**DOI:** 10.1007/s00063-024-01176-2

**Published:** 2024-09-06

**Authors:** Natascha Majunke, Dan Philipp, Lorenz Weidhase, Bastian Pasieka, Kevin Kunz, Frank Seidel, Robert Scharm, Sirak Petros

**Affiliations:** Interdisziplinäre Internistische Intensivmedizin, Universitätsklinikum Leipzig, Liebigstr. 20, 04103 Leipzig, Germany

**Keywords:** Fluid challenge, Passive leg raising test, Cardiac output, Preload responsiveness, Shock, Sepsis, Volumenchallenge, Passive-leg-raising-Test, Volumenreagibilität, Schock, Sepsis

## Abstract

**Background:**

The passive leg raising (PLR) test is a simple test to detect preload responsiveness. However, variable fluid doses and infusion times were used in studies evaluating the effect of PLR. Studies showed that the effect of fluid challenge on hemodynamics dissipates in 10 min. This prospective study aimed to compare PLR and a rapid fluid challenge (RFC) with a 300-ml bolus infused within 5 min in adult patients with a hemodynamic compromise.

**Materials and methods:**

Critically ill medical patients with signs of systemic hypoperfusion were included if volume expansion was considered. Hemodynamic status was assessed with continuous measurements of cardiac output (CO), when possible, and mean arterial pressure (MAP) at baseline, during PLR, and after RFC.

**Results:**

A total of 124 patients with a median age of 65.0 years were included. Their acute physiology and chronic health evaluation (APACHE) II score was 19.7 ± 6.0, with a sequential organ failure assessment (SOFA) score of 9.0 ± 4.4. Sepsis was diagnosed in 73.3%, and 79.8% of the patients were already receiving a norepinephrine infusion. Invasive MAP monitoring was established in all patients, while continuous CO recording was possible in 42 patients (33.9%). Based on CO changes, compared with those with RFC, the false positive and false negative rates with PLR were 21.7 and 36.8%, respectively, with positive and negative predictive values of 70.6 and 72.0%, respectively. Based on MAP changes, compared with those with RFC, the false positive and false negative rates with PLR compared to RFC were 38.2% and 43.3%, respectively, with positive and negative predictive values of 64.4 and 54.0%, respectively.

**Conclusion:**

This study demonstrated a moderate agreement between PLR and RFC in hemodynamically compromised medical patients, which should be considered when testing preload responsiveness.

## Abstract (de)

**Hintergrund:**

Der Passive-leg-raising(PLR)-Test ist eine einfache Methode zur Detektion der Volumenreagibilität. Studien zur Evaluation des PLR-Tests variieren allerdings hinsichtlich der Infusionsmengen und Infusionsdauer. Wie Untersuchungen zeigen, klingt der hämodynamische Effekt einer Volumen-Challenge innerhalb von 10 min ab. Ziel der vorliegenden prospektiven Studie an erwachsenen Patienten mit hämodynamischer Instabilität war es, den PLR-Test und eine rasche Volumen-Challenge („rapid fluid challenge“ [RFC]; 300 ml-Bolus verabreicht innerhalb von 5 min) zu vergleichen.

**Material und Methoden:**

Kritisch kranke internistische Patienten mit Zeichen einer systemischen Minderperfusion wurden eingeschlossen, wenn eine Volumengabe in Betracht kam. Der hämodynamische Status wurde initial sowie während PLR-Test und RFC soweit möglich anhand des Herzzeitvolumens (HZV) und des mittleren arteriellen Blutdrucks („mean arterial pressure“ [MAP]) erfasst.

**Ergebnisse:**

Insgesamt 124 Patienten wurden eingeschlossen (medianes Alter 65,0 Jahre, Acute-Physiology-And-Chronic-Health-Evaluation[APACHE]-II-Score 19,7 ± 6,0, Sequential-Organ-Failure-Assessment[SOFA]-Score 9,0 ± 4,4). Eine Sepsis lag bei 73,3 % der Patienten vor, und 79,8 % erhielten bereits eine Noradrenalininfusion. Eine invasive MAP-Überwachung war bei allen Patienten etabliert, eine kontinuierliche HZV-Messung war bei 42 Patienten (33,9 %) möglich. Auf Basis der HZV-Dynamik betrug die falsch-positive bzw. falsch-negative Rate mit PLR-Test im Vergleich zu RFC 21,7 % bzw. 36,8 %, mit positivem bzw. negativem prädiktivem Wert von 70,6 % bzw. 72,0 %. Mit Blick auf die MAP-Dynamik betrug die falsch-positive bzw. falsch-negative Rate mit PLR-Test im Vergleich zu RFC 38,2 % bzw. 43,3 %, mit positivem bzw. negativem prädiktivem Wert von 64,4 % bzw. 54,0 %.

**Schlussfolgerung:**

Diese Studie zeigte eine moderate Übereinstimmung zwischen PLR-Test und RFC bei hämodynamisch instabilen internistischen Patienten, die bei der Volumenreagibilitätstestung berücksichtigt werden sollte.

## Introduction

Fluid resuscitation is a frequent measure used to restore hemodynamic stability [23]. The passive leg raising (PLR) test is a simple and dynamic method and is one of the recommended tests to assess fluid responsiveness in critically ill patients [7]. Jabot et al. reported that a significant increase in cardiac output (CO) during PLR is observed when an average of 312 ml of blood is mobilized from the lower extremities [10]. However, published studies on PLR in comparison to exogenous fluid challenge used different amounts of fluid and/or different infusion times, which may compromise a general comparison of the results [19, 22, 28, 31]. The effect of a fluid challenge on hemodynamics dissipates within 10 min [2]. Therefore, the infusion time can affect fluid response [29].

A positive cardiac preload response to PLR or fluid challenge has been defined as an increase in CO of at least 10 or 15% [6, 18], with an increase in CO of at least 10% considered to be the best cutoff [18]. However, continuous CO monitoring may not be possible or feasible in every case. Septic shock is the most common type of shock [30] and international guidelines recommend a mean arterial pressure (MAP) target during hemodynamic resuscitation [7]. Therefore, MAP still plays a role as a monitoring tool despite its limitations. In a study on mechanically ventilated patients using PLR, responders showed a mean increase in MAP from 69 to 78 mm Hg, which is an increase by approximately 13% [19]. Another study using a fluid infusion in patients with acute circulatory failure reported a 13% increase in MAP as the best cutoff to detect a significant increase in CO with a positive predictive value of 82% [17].

Based on the above observations, the authors aimed to prospectively compare the hemodynamic effects of PLR and a rapid fluid challenge (RFC) in critically ill medical patients with signs of hemodynamic compromise using continuous CO and MAP measurements. Since the effect of PLR is immediate, it was hypothesized that a fluid bolus of 300 ml administered within 5 min in adults may allow a physiologically acceptable comparison with PLR. The major study objective was to assess the agreement between PLR and RFC regarding changes in CO and MAP.

## Patients and methods

This was a prospective study on critically ill medical patients with hemodynamic instability admitted to the medical intensive care unit (ICU) of a university hospital between September 2019 and September 2021. The study protocol was approved by the local ethics commission and conducted according to good clinical practice. The study conformed to the provisions of the Declaration of Helsinki (as revised in 2013).

Patients were included in the study after written informed consent. If a patient was not able to give consent and a legal guardian not yet available, study inclusion was achieved after a physician not belonging to the ICU team confirmed the urgency and validity of the study. Informed consent was then obtained once the patient or the legal guardian were able to do so. If a patient could not be included in the study, hemodynamic resuscitation was conducted according to the protocol of the unit that allows physicians to either apply PLR or an exogenous fluid challenge based on their preferences.

**Fig. 1:**
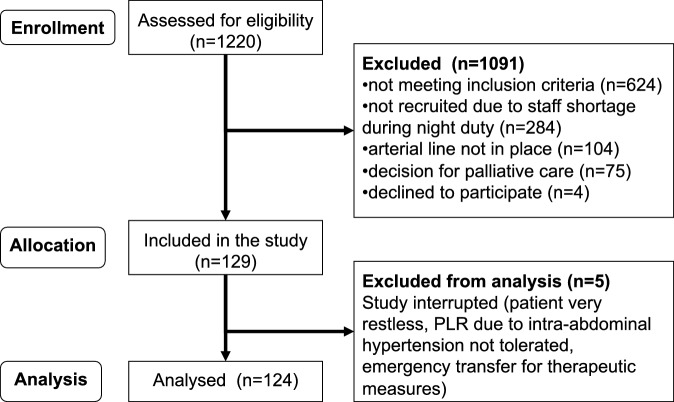
Patient recruitment flow diagram

The inclusion criteria were hemodynamic instability defined as a MAP <65 mm Hg or the need for vasopressor support to maintain a MAP >65 mm Hg and/or blood lactate >2 mmol/l plus the decision by the intensivist that testing for fluid responsiveness was justified. The exclusion criteria were hemorrhagic shock, irreversible brain damage, pregnancy and lactation as well as age <18 years (Fig. 1).

Continuous invasive arterial blood pressure measurement was established on every patient. Leveling and zeroing the transducer were carried out as described in the literature [26]. Continuous CO measurements were conducted with the PiCCO System (Pulsion Medical Systems, Feldkirchen, Germany) whenever this could be established without delay. CO is reported as cardiac index in l/min/m^2^.

A positive preload response to PLR and RFC was defined as an increase in CO of ≥ 10%. An increase in MAP of ≥ 13% was used to define a positive preload response. Where continuous CO measurements could not be established, only changes in MAP were used to evaluate preload response.

Baseline hemodynamic variables were documented immediately before the test. For patients on mechanical ventilation, ventilator settings and sedation dose were left unchanged during the test. Patients on controlled ventilation mode were managed with low tidal volume ventilation, which is the standard procedure of the unit. PLR was conducted according to published recommendations [21]. After the effect of PLR on CO and MAP had subsided, baseline hemodynamic variables were again recorded prior to RFC with the patient in the semi-recumbent position. Regardless of the results of the PLR, RFC was carried out with 300 ml of Ringer acetate solution as a bolus within 5 min. Hemodynamic variables were then recorded immediately after the fluid bolus. The changes in CO and MAP following PLR and RFC are given in percent of the baseline data recorded immediately before PLR and RFC, respectively.

The following variables were also recorded: age, gender, body weight (in kg), major reason for ICU admission, acute physiology and chronic health evaluation (APACHE) ΙΙ score, sequential organ failure assessment (SOFA) score, the need for mechanical ventilation and sedatives, the Richmond agitation sedation scale (RASS), and vasopressor dose for those on such a support.

The statistical analysis was performed with SPSS for Windows version 27 (IBM, Chicago, IL, USA) and GraphPad Prism version 9.3.1 (San Diego, CA, USA). Metric data were tested for normality of distribution using the Shapiro–Wilk test and presented as either mean with standard deviation or median [25–75 percentile]. Metric variables were compared using the Student t‑test or the Mann–Whitney U test depending on normality of distribution. Categorical variables were tested with the chi square test or Fisher exact test. The sensitivity and specificity as well as positive and negative predictive values of PLR compared to RFC were calculated. Taking patients with an RFC-induced increase in CO of ≥ 10% as responders and <10% as non-responders for binary classification, a receiver operating characteristics (ROC) analysis (± standard error of mean, SE) was conducted to assess the PLR-induced changes in CO as well as the changes in MAP following RFC. The optimal cutoff was estimated using the Youden index (= sensitivity + specificity −1). A *p*-value of < 0.05 was considered statistically significant.

## Results

A total of 1220 patients were screened for eligibility and 124 included in the study. The patient recruitment flow diagram is shown in Fig. 1. Most of the patients presented with sepsis (Table 1). The baseline clinical and laboratory data are shown in Table 2. Less than a third of the patients were on controlled mode of mechanical ventilation.

**Table 1:** General characteristics of the study population

Parameter	
*Age, years*	65.0 [55.0–74.8]
*Males (%)*	64.5
*Body weight (kg)*	80.0 [70.0–92.8]
*APACHE-II score*	19.7 ± 6.0
*SOFA score on the study day*	9.0 ± 4.4
*Sepsis (%)*	73.3
*Major admission reason (%)*	
Pulmonary	24.0
Gastrointestinal + hepatobiliary	20.8
Cardiovascular	18.4
Urogenital	10.4
Hematological	9.6
Others	16.8

Metric variables are given either as mean ± standard deviation or median with 25 and 75 percentiles in square brackets

*APACHE* acute physiology and chronic health evaluation; *SOFA* sequential organ failure assessment

**Table 2:** Baseline clinical and laboratory data of the study population

*MAP (mm Hg)*	63.4 ± 12.0
*Arterial lactate (mmol/l)*	2.4 [1.3–4.0]
*Invasive ventilation (%)*	63.6
Volume-controlled mode (%)	30.0
Pressure-assisted mode (%)	33.6
FiO_2_	0.30 [0.25–0.40]
PEEP (cm H_2_O)	8.0 [8.0–10.0]
*RASS (points)*	−2.0 [−4.0–0.0]
*Sedation requirement (%)*	56.9
*Vasopressor requirement (%)*	79.8
Norepinephrine dose (µg/kg/min)	0.23 [0.13–0.38]

Metric variables are given as mean ± standard deviation or median with 25 and 75 percentiles in square brackets

*MAP* mean arterial pressure; *FiO*_*2*_ fractional of inspired oxygen; *PEEP* positive end-expiratory pressure; *RASS* Richmond agitation sedation scale

Continuous CO recording could be established in 42 patients (33.9%). The changes in CO and MAP for responders and non-responders for both PLR and RFC are shown in Table 3. Based on the CO cutoff, 40.5% of the patients were PLR responders, while 46.3% were RFC responders. Comparing the response with PLR to that with RFC, 28.0% of the PLR non-responders became RFC responders, while 29.4% of the PLR responders were RFC non-responders (*p* = 0.011). Compared to RFC, the false positive and false negative rates with PLR were 21.7 and 36.8%, respectively, with positive and negative predictive values of 70.6 and 72.0%, respectively.

**Table 3:** Hemodynamic variables during the passive leg raising test and the rapid fluid challenge

	Passive leg raising		*p*-Value	Rapid fluid challenge		*p*-Value
*Co-based*	*Responders (N* *=* *17)*	*Non-responders (N* *=* *25)*	–	*Responders (N* *=* *19)*	*Non-responders (N* *=* *23)*	–
Baseline (l/m^2^/min)	2.8 ± 1.2	3.3 ± 1.8	0.27	2.6 ± 1.1	3.6 ± 1.8	0.03
Test end (l/m^2^/min)	3.3 ± 1.3	3.3 ± 1.7	–	3.2 ± 1.2	3.7 ± 1.8	–
Change from baseline (%)	16.6 [14.1–25.7]	0.0 [−5.1–4.9]	<0.001	21.5 [15.0–28.9]	0.5 [−1.2–3.2]	<0.001
*MAP-based*	*Responders (N* *=* *60)*	*Non-responders (N* *=* *64)*	–	*Responders (N* *=* *68)*	*Non-responders (N* *=* *56)*	–
Baseline (mm Hg)	62.6 ± 13.1	65.9 ± 14.9	0.18	59.6 ± 10.7	69.9 ± 16.7	<0.001
Test end (mm Hg)	79.9 ± 15.9	66.8 ± 15.1	–	77.3 ± 13.3	71.1 ± 15.2	–
Change from baseline (%)	23.3 [17.3–32.2]	3.4 [−3.6–9.2]	<0.001	29.3 [19.4–39.2]	3.6 [0.0–8.8]	<0.001

Test end describes the variables while the legs are elevated or immediately after the infusion of the bolus fluid

*CO* cardiac output given cardiac index in l/m^2^/min; *MAP* mean arterial blood pressure

The ROC analysis showed that a CO increase by ≥ 10.2% with PLR was the best cutoff to detect fluid responsiveness with an area under the curve (AUC) of 0.73 ± 0.08 (95% confidence interval 0.58–0.89), with a sensitivity of 63.2% and specificity of 78.3%.

Based on the MAP cutoff, 48.4% of the patients were PLR responders, while 54.9% were RFC responders. Comparing the response with PLR to that with RFC, 46% of the PLR non-responders became RFC responders, while 35.6% of the PLR responders were RFC non-responders (*p* = 0.047). Compared to RFC, the false positive and false negative rates with PLR were 38.2% and 43.3%, respectively. The positive and negative predictive values of PLR compared to RFC were 64.4% and 54.0%, respectively.

The ROC analysis showed that an MAP increase by ≥ 12.5% with RFC was the best cutoff to detect fluid responsiveness with an AUC of 0.65 ± 0.08 (95% confidence interval 0.48–0.82), with a sensitivity of 68.4% and specificity of 69.6%.

## Discussion

This prospective study showed a moderate agreement between the PLR test and an RFC in hemodynamically unstable medical patients. The reasons for discrepancies with regard to published results may be multifactorial. First, the amount of fluid administered to test fluid responsiveness in the literature was heterogeneous, ranging from 250 to 1000 ml [14, 29]. The authors administered 300 ml based on the findings of Jabot et al. [10]. A recent study reported that a fluid bolus of 4 ml/kg body weight would reliably detect responders and non-responders [1]. Another recent study also reported that 250 ml fluid with a threshold of 9.6% increase in stroke volume showed the highest accuracy in detecting fluid responsiveness in patients with shock [3]. Second, the duration of fluid infusion also varies in the literature. Previous studies reported infusion times of up to 30 min [14, 29], while this was only 5 min in the current study.

The results of the ROC analysis in this study are in agreement with previous publications for both PLR compared to exogenous fluid challenge [18] as well as for an increase in MAP compared to that in cardiac output [17].

Pain or discomfort should be avoided during PLR [21]. Nevertheless, critically ill patients may be restless, which may contribute to false positive results with PLR or make PLR impossible. Changes in heart rate as a sign of sympathetic activity during PLR may not be reliable enough [5]. In the current study, only 30% of the patients were on controlled ventilation mode and just over half of the patients were sedated, with a variable agitation and sedation state.

PLR may also be false negative, one possible reason being intra-abdominal hypertension (IAH), with an intra-abdominal pressure >15 mm Hg considered a limitation [13]. IAH also reduces the amplitude of the PLR-induced changes in cardiac output in fluid-responsive patients [4]. No clinical sign of such a marked IAH was observed in this study. However, intra-abdominal pressure was not measured to confirm this assumption. It is not fully clear why certain patients with IAH are fluid responsive, while others are not, although the PLR test even led to a decrease in intra-abdominal pressure [4]. Another possible cause of a false negative PLR test could be marked intravascular hypovolemia, so that the blood volume to be shifted from the lower extremities following PLR might have been insufficient. However, this possibility has not been systematically investigated. Furthermore, most of the patients were on a vasopressor infusion, which may have a dose-dependent influence on cardiac preload that may affect preload response to PLR [16]. The ventilator settings among the patients on mechanical ventilation in the current study could not have had a negative influence on the PLR test [20].

PLR can help avoid unnecessary fluid administration. However, holding back fluid resuscitation on the basis of a possibly false negative PLR may also be unfavorable. Fluid challenge is only a tool to test for cardiac preload response. The unresolved issue is the fluid dose to be administered following a positive preload challenge. This uncertainty may be a source of fluid overload that is associated with poor outcome [15].

The appropriate means to rapidly assess preload response is a challenge in daily patient care. Continuous CO monitoring is the standard method to detect fluid responsiveness. However, this may not be possible or easily established or feasible. The cost of such monitoring techniques should also be taken into consideration. Other dynamic techniques have their own limitations [20], and they may also not be easily available everywhere. Additionally, the shortage of qualified staff mastering these techniques should be considered. Nevertheless, lack of a CO measurement technique would be an unlikely argument to withhold testing for fluid responsiveness. A recent Danish survey showed that blood pressure is still the most frequently used trigger to evaluate fluid response in emergency departments [11]. International guidelines still recommend MAP as a target variable for hemodynamic resuscitation [7], with due consideration of its limitations [18, 20].

An increase in cardiac output does not necessarily translate into improvement of the microcirculation. Correct identification of the patients who may benefit from resuscitation in terms of microcirculatory improvement is still a challenge [24]. Clinical signs such as cold and pale extremities, capillary refill time or skin mottling may be useful to identify patients with systemic hypoperfusion. Normalization of the capillary refill time during hemodynamic resuscitation of patients with septic shock has been investigated [9]. However, inter-observer variations and patient heterogeneity may be relevant limitations for its use as a monitoring tool [27]. A recent study on healthy volunteers has also demonstrated variations depending on how capillary refill time is investigated [12]. The time span between fluid challenge and the detection of such microcirculatory changes may also be variable [8]. Furthermore, fluid responsiveness is time-dependent, with the hemodynamic effect lost within a short time in a proportion of patients [25]. Further studies are still required to identify objective, timely, reproducible and widely available parameters of tissue perfusion to monitor the effect of fluid resuscitation.

This study has certain limitations. First, it is monocentric and included only critically ill medical patients. However, the findings may contribute to our understanding of the routine management of the patient with acute circulatory dysfunction. Second, patients could not be included in the study during the night shifts due to staff shortage. It is not clear how far this could have been a challenge in similar studies, since this issue was not systematically addressed. However, the authors believe that this should not have significantly affected the conclusion, since every patient was her or his own control. Third, continuous cardiac output measurements could not be performed in every patient. However, the authors believe that their observations show the real world acute patient care, in which cardiac output monitoring may not be immediately available or feasible for various reasons. Such techniques are usually reserved for the severely ill patients in need of complex and protracted hemodynamic management. Fourth, intra-abdominal pressure was not measured unless this was justified based on clinical judgement. However, it is unlikely that patients with an IAH of > 15 mm Hg would be overlooked during a proper clinical examination.

## Conclusion

The present study showed moderate agreement between the passive leg raising test and a rapid fluid challenge with 300 ml fluid bolus assessed using both continuous cardiac output and mean arterial pressure measurements in critically ill medical patients, which should be considered when testing preload responsiveness.

## Declarations

All procedures performed in studies involving human participants or on human tissue were in accordance with the ethical standards of the institutional and/or national research committee and with the 1975 Helsinki declaration and its later amendments or comparable ethical standards. Informed consent was obtained from all individual participants included in the study. ClinicalTrials.gov ID: NCT04089098.

## References

[1] Aya HD, Rhodes A, Chis Ster I et al (2017) Hemodynamic Effect of Different Doses of Fluids for a Fluid Challenge: A Quasi-Randomized Controlled Study. Crit Care Med 45:e161–e168 27655325 10.1097/CCM.0000000000002067

[2] Aya HD, Ster IC, Fletcher N et al (2016) Pharmacodynamic Analysis of a Fluid Challenge. Crit Care Med 44:880–891 26683506 10.1097/CCM.0000000000001517

[3] Barthelemy R, Kindermans M, Delval P et al (2022) Accuracy of cumulative volumes of fluid challenge to assess fluid responsiveness in critically ill patients with acute circulatory failure: a pharmacodynamic approach. Br J Anaesth 128:236–243 34895718 10.1016/j.bja.2021.10.049

[4] Beurton A, Teboul JL, Girotto V et al (2019) Intra-Abdominal Hypertension Is Responsible for False Negatives to the Passive Leg Raising Test. Crit Care Med 47:e639–e647 31306258 10.1097/CCM.0000000000003808

[5] Caille V, Jabot J, Belliard G et al (2008) Hemodynamic effects of passive leg raising: an echocardiographic study in patients with shock. Intensive Care Med 34:1239–1245 18351322 10.1007/s00134-008-1067-y

[6] Cherpanath TG, Hirsch A, Geerts BF et al (2016) Predicting Fluid Responsiveness by Passive Leg Raising: A Systematic Review and Meta-Analysis of 23 Clinical Trials. Crit Care Med 44:981–991 26741579 10.1097/CCM.0000000000001556

[7] Evans L, Rhodes A, Alhazzani W et al (2021) Surviving sepsis campaign: international guidelines for management of sepsis and septic shock 2021. Crit Care Med 49:e1063–e1143 34605781 10.1097/CCM.0000000000005337

[8] Hernandez G, Luengo C, Bruhn A et al (2014) When to stop septic shock resuscitation: clues from a dynamic perfusion monitoring. Ann Intensive Care 4:30 25593746 10.1186/s13613-014-0030-z PMC4273696

[9] Hernandez G, Ospina-Tascon GA, Damiani LP et al (2019) Effect of a resuscitation strategy targeting peripheral perfusion status vs serum lactate levels on 28-day mortality among patients with septic shock: the ANDROMEDA-SHOCK randomized clinical trial. JAMA 321:654–664 30772908 10.1001/jama.2019.0071 PMC6439620

[10] Jabot J, Teboul JL, Richard C et al (2009) Passive leg raising for predicting fluid responsiveness: importance of the postural change. Intensive Care Med 35:85–90 18795254 10.1007/s00134-008-1293-3

[11] Jessen MK, Simonsen BY, Thomsen MH et al (2022) Fluid management of emergency department patients with sepsis—A survey of fluid resuscitation practices. Acta Anaesthesiol Scand 66:1237–1246 36054552 10.1111/aas.14141 PMC9805143

[12] La Via L, Sanfilippo F, Continella C et al (2023) Agreement between Capillary Refill Time measured at Finger and Earlobe sites in different positions: a pilot prospective study on healthy volunteers. BMC Anesthesiol 23:30 36653739 10.1186/s12871-022-01920-1 PMC9847031

[13] Mahjoub Y, Touzeau J, Airapetian N et al (2010) The passive leg-raising maneuver cannot accurately predict fluid responsiveness in patients with intra-abdominal hypertension. Crit Care Med 38:1824–1829 20639753 10.1097/CCM.0b013e3181eb3c21

[14] Messina A, Calabro L, Pugliese L et al (2022) Fluid challenge in critically ill patients receiving haemodynamic monitoring: a systematic review and comparison of two decades. Crit Care 26:186 35729632 10.1186/s13054-022-04056-3 PMC9210670

[15] Messmer AS, Zingg C, Muller M et al (2020) Fluid overload and mortality in adult critical care patients—a systematic review and meta-analysis of observational studies. Crit Care Med 48:1862–1870 33009098 10.1097/CCM.0000000000004617

[16] Monnet X, Jabot J, Maizel J et al (2011) Norepinephrine increases cardiac preload and reduces preload dependency assessed by passive leg raising in septic shock patients. Crit Care Med 39:689–694 21263328 10.1097/CCM.0b013e318206d2a3

[17] Monnet X, Letierce A, Hamzaoui O et al (2011) Arterial pressure allows monitoring the changes in cardiac output induced by volume expansion but not by norepinephrine. Crit Care Med 39:1394–1399 21336124 10.1097/CCM.0b013e31820edcf0

[18] Monnet X, Marik P, Teboul JL (2016) Passive leg raising for predicting fluid responsiveness: a systematic review and meta-analysis. Intensive Care Med 42:1935–1947 26825952 10.1007/s00134-015-4134-1

[19] Monnet X, Rienzo M, Osman D et al (2006) Passive leg raising predicts fluid responsiveness in the critically ill. Crit Care Med 34:1402–1407 16540963 10.1097/01.CCM.0000215453.11735.06

[20] Monnet X, Shi R, Teboul JL (2022) Prediction of fluid responsiveness. What’s new? Ann Intensive Care 12:46 35633423 10.1186/s13613-022-01022-8 PMC9148319

[21] Monnet X, Teboul JL (2015) Passive leg raising: five rules, not a drop of fluid! Crit Care 19:18 25658678 10.1186/s13054-014-0708-5 PMC4293822

[22] Muller L, Toumi M, Bousquet PJ et al (2011) An increase in aortic blood flow after an infusion of 100 ml colloid over 1 minute can predict fluid responsiveness: the mini-fluid challenge study. Anesthesiology 115:541–547 21792056 10.1097/ALN.0b013e318229a500

[23] Myburgh JA, Mythen MG (2013) Resuscitation fluids. N Engl J Med 369:1243–1251 24066745 10.1056/NEJMra1208627

[24] Pinsky MR, Cecconi M, Chew MS et al (2022) Effective hemodynamic monitoring. Crit Care 26:294 36171594 10.1186/s13054-022-04173-z PMC9520790

[25] Roger C, Zieleskiewicz L, Demattei C et al (2019) Time course of fluid responsiveness in sepsis: the fluid challenge revisiting (FCREV) study. Crit Care 23:179 31097012 10.1186/s13054-019-2448-z PMC6524325

[26] Saugel B, Kouz K, Meidert AS et al (2020) How to measure blood pressure using an arterial catheter: a systematic 5‑step approach. Crit Care 24:172 32331527 10.1186/s13054-020-02859-w PMC7183114

[27] Sheridan DC, Baker SD, Kayser SA et al (2017) Variability of Capillary Refill Time among Physician Measurements. J Emerg Med 53:e51–e57 28941555 10.1016/j.jemermed.2017.06.035

[28] Smorenberg A, Cherpanath TGV, Geerts BF et al (2018) A mini-fluid challenge of 150 mL predicts fluid responsiveness using Modelflow(R) pulse contour cardiac output directly after cardiac surgery. J Clin Anesth 46:17–22 29367093 10.1016/j.jclinane.2017.12.022

[29] Toscani L, Aya HD, Antonakaki D et al (2017) What is the impact of the fluid challenge technique on diagnosis of fluid responsiveness? A systematic review and meta-analysis. Crit Care 21:207 28774325 10.1186/s13054-017-1796-9 PMC5543539

[30] Vincent JL, De Backer D (2013) Circulatory shock. N Engl J Med 369:1726–1734 24171518 10.1056/NEJMra1208943

[31] Vincent JL, Weil MH (2006) Fluid challenge revisited. Crit Care Med 34:1333–1337 16557164 10.1097/01.CCM.0000214677.76535.A5

